# Breaking the Recycling Bottleneck of Thermosets via Bio‐Tailoring Technology

**DOI:** 10.1002/advs.202501617

**Published:** 2025-05-08

**Authors:** Jinping Yu, Huan Wang, Yang Xu, Boutrous Wemegah, Yang Lan, Jing Li, Li Yang, Guanjun Chang

**Affiliations:** ^1^ State Key Laboratory of Environment‐Friendly Energy Materials and School of Materials and Chemistry Southwest University of Science and Technology Mianyang 621010 P. R. China; ^2^ Centre for Nature‐Inspired Engineering Department of Chemical Engineering University College London London WC1E 7JE UK; ^3^ Research Center of Laser Fusion China Academy of Engineering Physics Mianyang 621900 P. R. China; ^4^ Department of Chemical and Biomolecular Engineering University of Pennsylvania Philadelphia PA 19104 USA

**Keywords:** bio‐tailoring technology, eco‐friendliness, recycling, thermosets

## Abstract

Thermosets present significant recycling challenges due to irreversible chemical crosslinking, resulting in resource waste and environmental pollution. This paper introduces a new strategy for designing thermosets with excellent recycling properties driven by **Bio‐Tailoring** technology, achieving amazing green sustainable development. These thermosets are a class of infusible and insoluble polymers owing to chemical crosslinking. The indole groups in the cross‐linked network can be accurately identified and tailored by microorganisms, giving rise to the cross‐linked network being tailored into new linear polymers and small molecule segments, named the **Bio‐Tailoring** technology. Linear polymers can be used as a new plastic packaging material that involves high transparency and appreciable mechanical properties. Meanwhile, the cross‐linked small molecule moiety is a fluorescent functional unit with a unique push‐pull electronic structure that leads to color‐changing under external stimulation and can be applied to anti‐counterfeiting. Overall, this strategy provides an innovative solution for the sustainable recycling of thermosets and opens a new path for the environmental transformation of the plastics industry.

## Graphical abstract

Recyclable thermosets are achieved by using the **Bio‐Tailoring** technology. The **Bio‐Tailoring** technology effectively addresses the longstanding paradox surrounding the recyclable and reuse properties of thermosets, breaking the recycling bottleneck of thermosets.

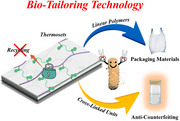

## 1 Introduction

Thermosets are widely used in aerospace, mechanical, and electronic applications because of their convenience and affordability to make and use.^[^ ^1^, ^2^, ^3^ ^]^ However, conventional thermosets present significant recycling challenges due to irreversible chemical crosslinking, leading to thermosets quickly becoming single‐use items, and only ≈14% are ultimately recycled.^[^ ^4^, ^5^, ^6^ ^]^ To reduce resource waste, the introduction of dynamic bonds in thermosets (covalent or non‐covalent) can greatly improve resource utilization. However, according to previous studies, the dissociation process of recyclable thermosets based on dynamic bonds requires specific additional stimuli (such as acids, bases, high temperatures, high pressures, and so on), and the recycling of these new thermosets relies on specific functional groups and high energy demands.^[^ ^7^, ^8^, ^9^, ^10^ ^]^ Meanwhile, another issue that cannot be ignored is apt to leave toxic byproducts during the recycling process.^[^ ^11^, ^12^, ^13^, ^14^, ^15^, ^16^ ^]^ Therefore, the development of innovative green recycling technologies for the sustainable management of thermosets is an intriguing and fascinating challenge.^[^ ^17^, ^18^, ^19^, ^20^, ^21^ ^]^ Recently, bio‐recycling technology has been increasingly recognized for its potential in resource recovery owing to sustainability and eco‐friendliness. This technology gently treats waste plastics by enzymatic reactions of microorganisms and promotes effective recycling of plastics.^[^ ^22^, ^23^, ^24^, ^25^ ^]^ Whereas, compared to the waste plastics generated by human activities, the amount that can be recycled is negligible by bio‐recycling technology.^[^ ^26^, ^27^, ^28^ ^]^ This can be attributed to that the biological enzymes reacting to plastics require long‐term strain discovery/evolution and complicated enzyme engineering. In addition, plastics will produce a series of short‐chain byproducts through bio‐recycling, which increases the separation cost.^[^ ^29^, ^30^, ^31^ ^]^ More importantly, most of the current bio‐recycling is still limited to bio‐based materials, and it has hardly been explored in the recycling of thermosets due to irreversible chemical crosslinking.^[^ ^32^, ^33^, ^34^, ^35^ ^]^ Consequently, investigating the design and preparation of readily accessible thermosets for effective recycling is essential for exploring new technologies for treating plastic waste and opening a new path for the environmental transformation of the plastics industry.

**Figure 1:**
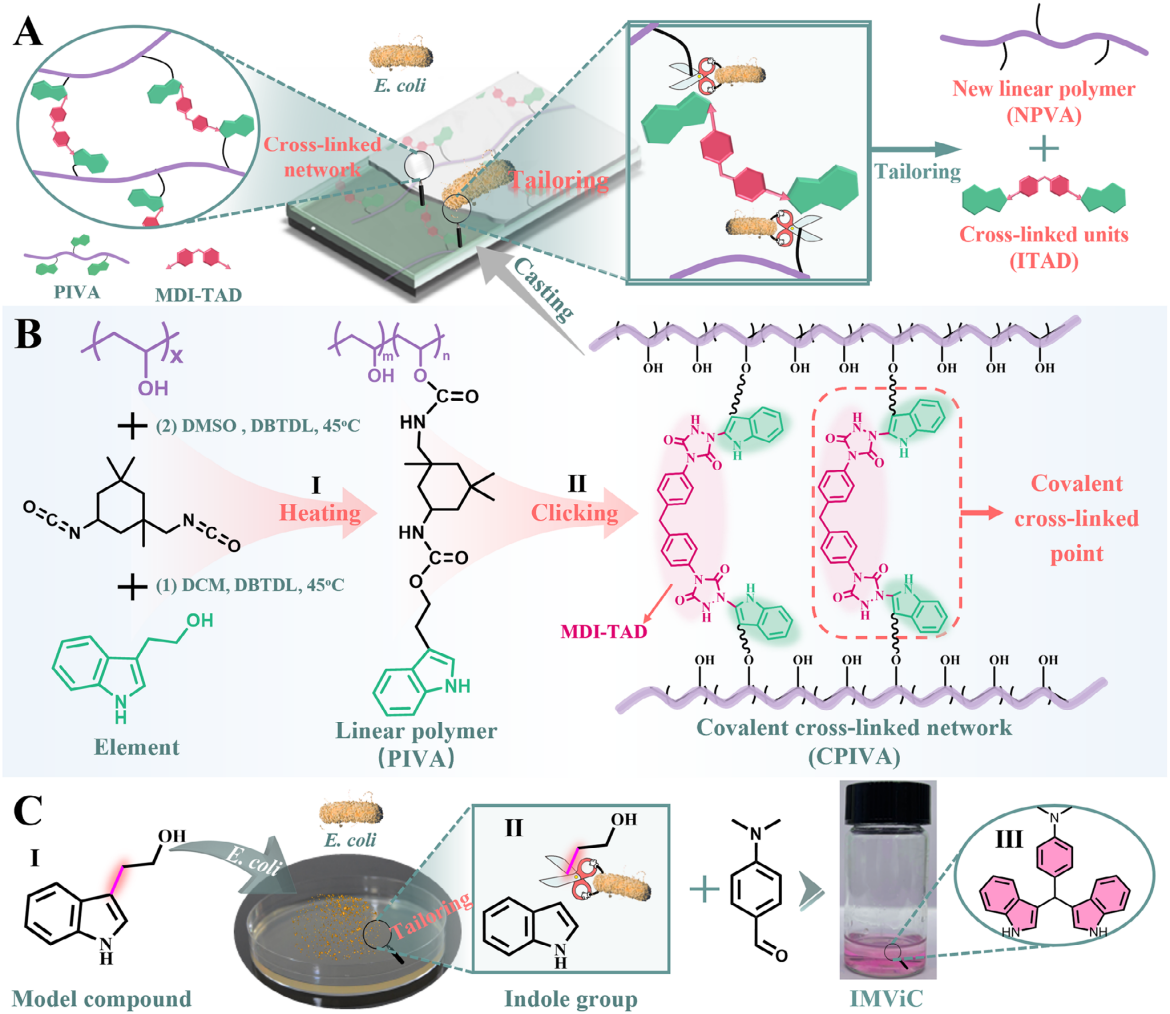
**Bio‐Tailoring** technology and synthesis of recyclable thermosets. A) The linear recycling of thermosets driven by the **Bio‐Tailoring** technology. B) The synthesis pathway of linear polymer chains PIVA (B‐I) and CPIVA thermosets (B‐II) (experimental procedure details are provided in Supporting Information). C) The biological reaction of small molecule compounds (Tryp) in *E. coli* (C‐I, II), and the positive indole reaction of liquid medium (C‐III).

In this work, we describe a new concept for designing thermosets with excellent recycling properties driven by **Bio‐Tailoring** technology, breaking the recycling bottleneck of thermosets. Herein, we employ the following strategy: the cross‐linked network will be tailored into new linear polymers and small molecule segments, named the **Bio‐Tailoring** technology (**Figure** **1**A). The linear polymers obtained can be used as a new plastic packaging material. Meanwhile, the resulting small molecule units will be applied to anti‐counterfeiting due to the unique push‐pull electronic structure, described as color‐changing under external stimulation. In contrast to traditional bio‐recycling, **Bio‐Tailoring** technology has the advantages of simple operation and flexible treatment, which promotes the sustainable reuse of thermosets and the development of the circular economy. These studies will help us to gain a deeper insight into the influences of **Bio‐Tailoring** technology on the construction of outstanding eco‐friendly thermosets.

## 2 Results and Discussion

### 2.1 Synthesis of Indole‐Based Recyclable Thermosets

To prove this concept, we have constructed a series of indole‐based recyclable thermosets. First, the linear polymer is synthesized by polyvinyl alcohol (PVA), isophorone diisocyanate (IPDI), and tryptophol (Tryp), which is known as PIVA (Figure 1B‐I). This polymer forms covalently cross‐linked thermosets by click reaction with MDI‐TAD (Figure 1B‐II). The synthesis of MDI‐TAD is carried out following our previously described procedure.^[^ ^36^ ^]^ The cross‐linked density of the thermosets can be readily manipulated by varying the feed ratio of MDI‐TAD to the polymers. The resulting thermosets are referred to as CPIVA‐x, where x indicates the molar fraction of MDI‐TAD in comparison to indole in the initial mixture. Characterization and experimental procedure details are provided in Supporting Information.

### 2.2 Bio‐Tailoring of Model Compounds

To shed light on the possible origin of the significant decomposed performance via **Bio‐Tailoring** technology, we chose *Escherichia coli* (*E. coli*) as a representative microorganism and studied the biological reaction of model compounds (Tryp) in *E. coli* (Figure 1C). The specific experimental procedures and the positive indole reaction are shown in Supporting Information.^[^ ^37^ ^]^ It is well known that indole can react quickly with 4‐dimethylami‐nobenzaldehyde in ether to form rose indole, which makes the solution red. This remarkable color change can be used as a rapid detection method for indole. The outcome of the positive indole reaction indicates that Tryp could also react with 4‐dimethylaminobenzaldehyde after being tailored by *E. coli*, resulting in the solution changing to red (Figure 1C‐III). The obvious color change suggests the presence of indole groups in the liquid medium. We reasonably describe this change process as follows (Figure 1C): The alkane branched chain at the indole C3‐position in Tryp is tailored by *E. coli*, exposing the indole C3‐position (Figure 1C‐I,II); Then the exposed indole C3‐position can be rapidly substituted with 4‐dimethylaminobenzaldehyde to form rose indole, bringing out the red solution (Figure 1C‐III). To further understand the mechanism of **Bio‐Tailoring Technology**, we carried out a nuclear magnetic comparison experiment of **Tryp**, **Tryp Compound** (the extracting solution of liquid medium before Bio‐Tailoring), and **Tailored Compound** (the extracting solution of the liquid medium after Bio‐Tailoring) (Figure S19, Supporting Information). The ^1^H‐NMR results showed that Tryp was tailored into small indole molecules by *E. coli*. More interestingly, we emphasize that this is only true when using **Bio‐Tailoring** technology as the way to the decomposition. The above results lay a theoretical foundation for the bio‐recycling of thermosets.

### 2.3 Crosslinking Behaviors of the Thermosets

**Figure 2:**
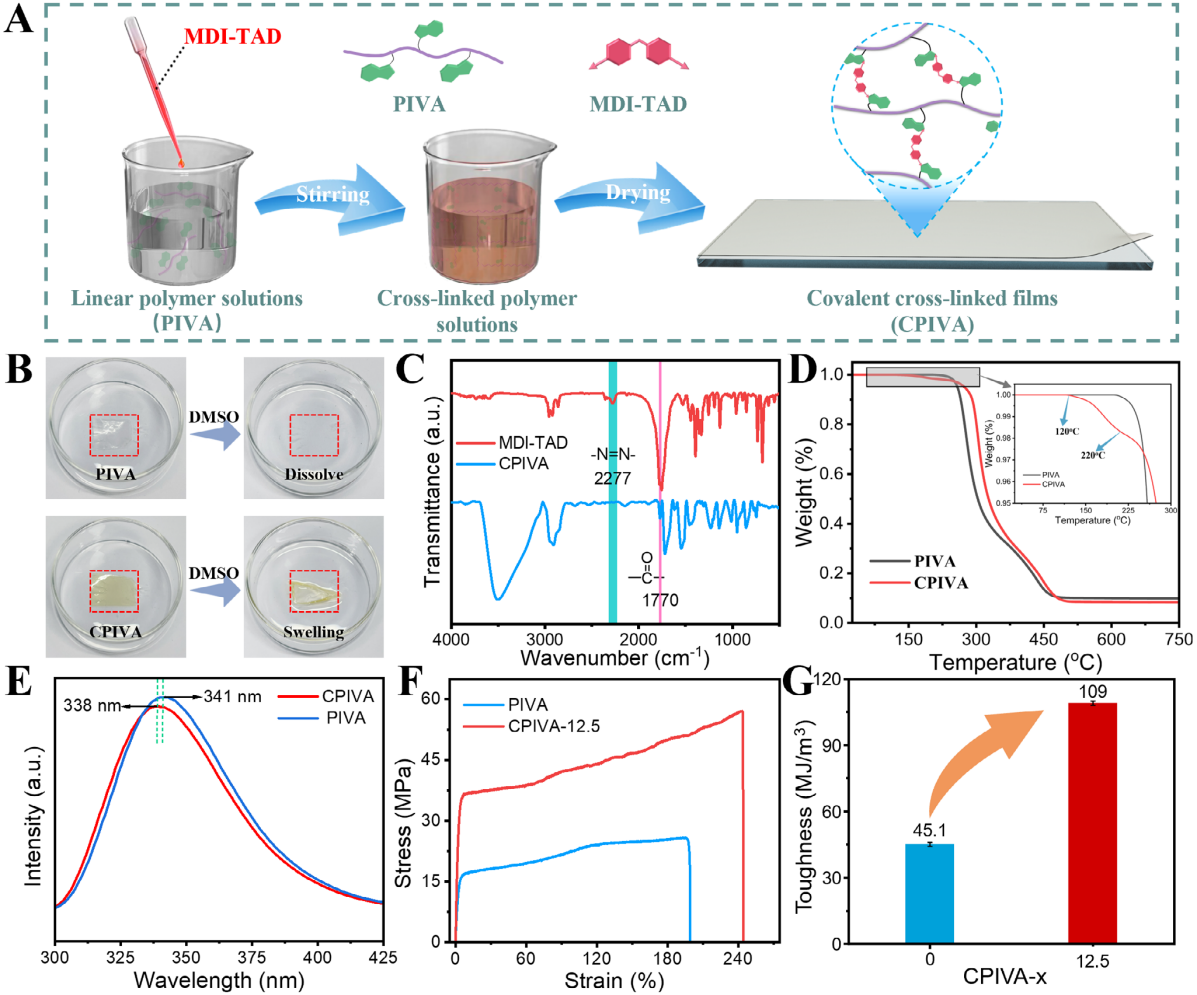
Crosslinking behaviors of the thermosets. A) Preparation process of CPIVA thermosets. B) The dissolution experiment of linear polymer chains PIVA and CPIVA thermosets. C) The structure of the prepared thermosets was characterized by FTIR. D) TG curves of linear polymer chains PIVA and CPIVA thermosets. E) Fluorescence spectra of CPIVA (red line) and PIVA (blue line). F) Stress‐strain curves of linear polymer chains PIVA and CPIVA‐12.5 plastic. G) Tensile toughness of linear polymer chains PIVA and CPIVA‐12.5 plastic.

The corresponding thermoset films are prepared using the solvent‐casting method (**Figure** **2**A). At room temperature, the CPIVA films are transparent, non‐tacky yellow solid (Figure S10, Supporting Information). Compared with the rapid dissolution of PIVA, the CPIVA films only slightly curl and swell, verifying the existence of crosslinking (Figure 2B; Table S1, Supporting Information). Subsequently, the structure of the CPIVA films are characterized by FTIR. The disappearance of the ─N═N─ stretching vibration peak at 2277 cm^−1^ indicates that there is no unreacted MDI‐TAD in CPIVA, and the appearance of the characteristic carbonyl stretching vibration peak at 1770 cm^−1^ demonstrates the successful introduction of MDI‐TAD into polymer networks (Figure 2C). Thermogravimetry analysis (TG) curve has a decomposition peak at 120 °C, which is the result of the decomposition of TAD at elevated temperatures (Figure 2D).^[^ ^38^ ^]^ It is well known that the click reaction of indole and TAD is competitive. In previous reports, it has been confirmed that TAD first undergoes a click reaction with the C2‐position of indole and does not destroy the aromaticity of indole.^[^ ^36^, ^38^ ^]^ These results can be confirmed by the weak change in the fluorescence intensity of the polymer solution (Figure 2E) and the retention of the UV absorption peak before and after crosslinking, respectively (Figure S15, Supporting Information). Moreover, uniaxial tensile tests reveal an obvious enhancement in the mechanical properties of the CPIVA because of the covalent crosslinking (Figure 2F; Figure S13, Supporting Information). The dynamic net effect exhibits an approximately twofold increase in tensile strength, escalating from σ PIVA = 26 MPa to σ CPIVA‐12.5 = 57 MPa. However, the covalent crosslinking limits the mobility of the polymer chains, and the macroscopic performance is a slight increase in elongation at break (from 200% to 243%). Furthermore, there is a remarkable 2.5‐fold enhancement in tensile toughness, advancing from 45.1 to 109 MJ m^−3^ (Figure 2G). This room‐temperature fast‐click crosslinking method not only offers controllable crosslinking, and simple operation but also enables the fabrication of thermosets with excellent mechanical properties. In addition, we anticipate that these thermosets can be efficiently recycled and reused through **Bio‐Tailoring** technology, enhancing their sustainability, as mentioned later.

### 2.4 Recycling Behaviors of the Thermosets

**Figure 3:**
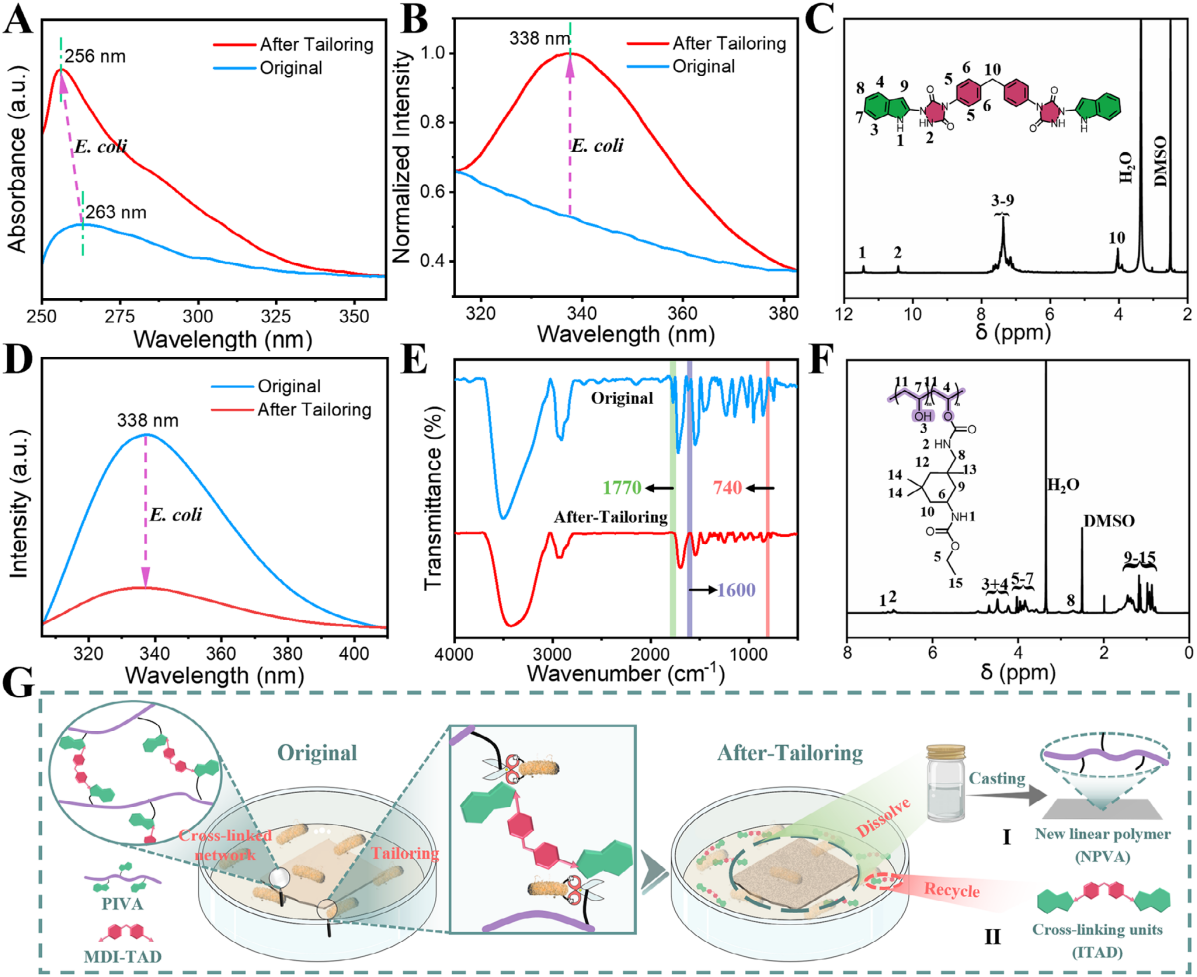
Bio‐recycling behaviors of the CPIVA plastics. UV absorption spectra A) and fluorescence spectra B) of the liquid medium before and after tailoring CPIVA‐12.5 plastic. C) ^1^H‐NMR spectra of the liquid medium after tailoring CPIVA‐12.5 plastic. Fluorescence spectra D) and FTIR E) spectra of CPIVA‐12.5 plastic before and after tailoring. F) ^1^H‐NMR spectra of CPIVA‐12.5 plastic after tailoring. G) The specific **Bio‐Tailoring** mechanism of *E. coli*.

The **Bio‐Tailoring** technology can endow excellent recycling performance of CPIVA thermosets. We conduct a series of experiments to explain this unprecedented phenomenon. Here, we chose the CPIVA‐12.5 plastic as a representative example to demonstrate our hypothesis. First, CPIVA‐12.5 plastic (6 cm × 3 cm, 0.05 mm thick) is immersed in *E. coli* liquid medium. The changes in CPIVA‐12.5 plastic are monitored by recording the macroscopic phenomenon of the liquid medium and conducting the positive indole reaction (Figures S11 and S20, Supporting Information). The results of the positive indole reaction show that indole groups gradually presented in the liquid medium with the progress of **Bio‐Tailoring**. Compared with CPIVA‐12.5 plastic, the residual plastic in the liquid medium displays an amazing dissolution phenomenon after ≈50 days of **Bio‐Tailoring** (Figure S21, Supporting Information). To propose this microscopic change mechanism, we further characterize the liquid medium and residual plastics. **Figure** **3**A depicts the liquid medium's UV absorption spectra in the absence and presence of CPIVA‐12.5 plastic. As expected, the liquid medium that tailors CPIVA‐12.5 plastic produces a strong UV absorption peak at 256 nm, which is a classical *π*–*π* ^*^ transition absorption peak of the indole group (Figure 3A). Furthermore, the fluorescence spectra also indicate a strong fluorescence emission peak at 338 nm (Figure 3B). ^1^H‐NMR spectra of the liquid medium further reveal the above transformation (Figure 3C). The distinct characteristic H in the small molecule segments appears and is marked on the map: The ‐NH proton signal of the indole ring is observed at 11.43 ppm; The ─NH proton peaks of the triazolindione structure appear at 10.43 ppm; Additionally, the methylene group attached to the benzene ring exhibits a signal at 4.03 ppm. The ^1^H‐NMR spectra suggest that the small molecule segments (ITAD) are tailored from CPIVA‐12.5 plastic by *E. coli* and dropped into the liquid medium.

Based on this, the residual plastic is further characterized. First, the CPIVA‐12.5 plastic before and after tailoring is tested by fluorescence spectroscopy, and the spectra clearly exhibit that the fluorescence intensity at 338 nm is distinctly reduced (Figure 3D). Such findings are attributed to that CPIVA‐12.5 plastic has a large number of indole luminescent groups, and the indole groups gradually fall off with the tailoring process of *E. coli*, resulting in a significant decrease in the fluorescence intensity of the plastic. The FTIR spectra of CPIVA‐12.5 plastic before and after tailoring also obtained the same results (Figure 3E). First, the characteristic C─H out‐of‐plane deformation vibration peak in the benzopyrrole structure of indole at 740 cm^−1^ and the vibrational alteration of the benzene within the indole structure at 1600 cm^−1^ both disappear in the residual plastic. Second, the characteristic carbonyl stretching vibration peak at 1770 cm^−1^ in the TAD structure disappears in the residual plastic. Moreover, we carry out ^1^H‐NMR experiments of the residual plastic to further reveal the conversion above (Figure 3F). The ^1^H‐NMR spectra shows that the crosslinking network of CPIVA‐12.5 plastic is tailored into the new linear polymers and small molecule segments that fall from CPIVA‐12.5 plastic to liquid medium under the action of gravity. It is worth noting that this structural transformation is highly consistent with the variation law of the liquid medium mentioned above.

Considering the excellent recycling behaviors of CPIVA‐12.5 plastic, we propose an unprecedented bio‐recycling mechanism of thermosets, named **Bio‐Tailoring** technology. The specific **Bio‐Tailoring** technology is demonstrated as follows (Figure 3G). First, the indole functional units in the cross‐linked network can be accurately identified by *E. coli*, and the alkane branched chain at the indole C3‐position is tailored, resulting in the cross‐linked network being tailored into new linear polymers (NPVA) and small molecule segments (ITAD) (Figure 3G–I, II), described as the **Bio‐Tailoring** technology. Second, the ITAD is prone to separate from CPIVA‐12.5 plastic and drop into the liquid medium. The NPVA still retains a large fragmentation and floats in the liquid medium. For CPIVA‐12.5 plastic, the recycling and separation of thermosets are easily accomplished by **Bio‐Tailoring** technology, achieving green sustainable development. In addition, the NPVA dissolved in DMSO could be reconstructed into a film and utilized as a new plastic packaging material since the film still maintains an appreciable mechanical property (Figure S22, Supporting Information). Furthermore, the ITAD can be used as a functionalized precursor owing to the unique push‐pull electronic structure. This phenomenon is defined as linear recycling and functional reuse of thermosets via **Bio‐Tailoring** technology.

### 2.5 Functional Reuse of the Thermosets

**Figure 4:**
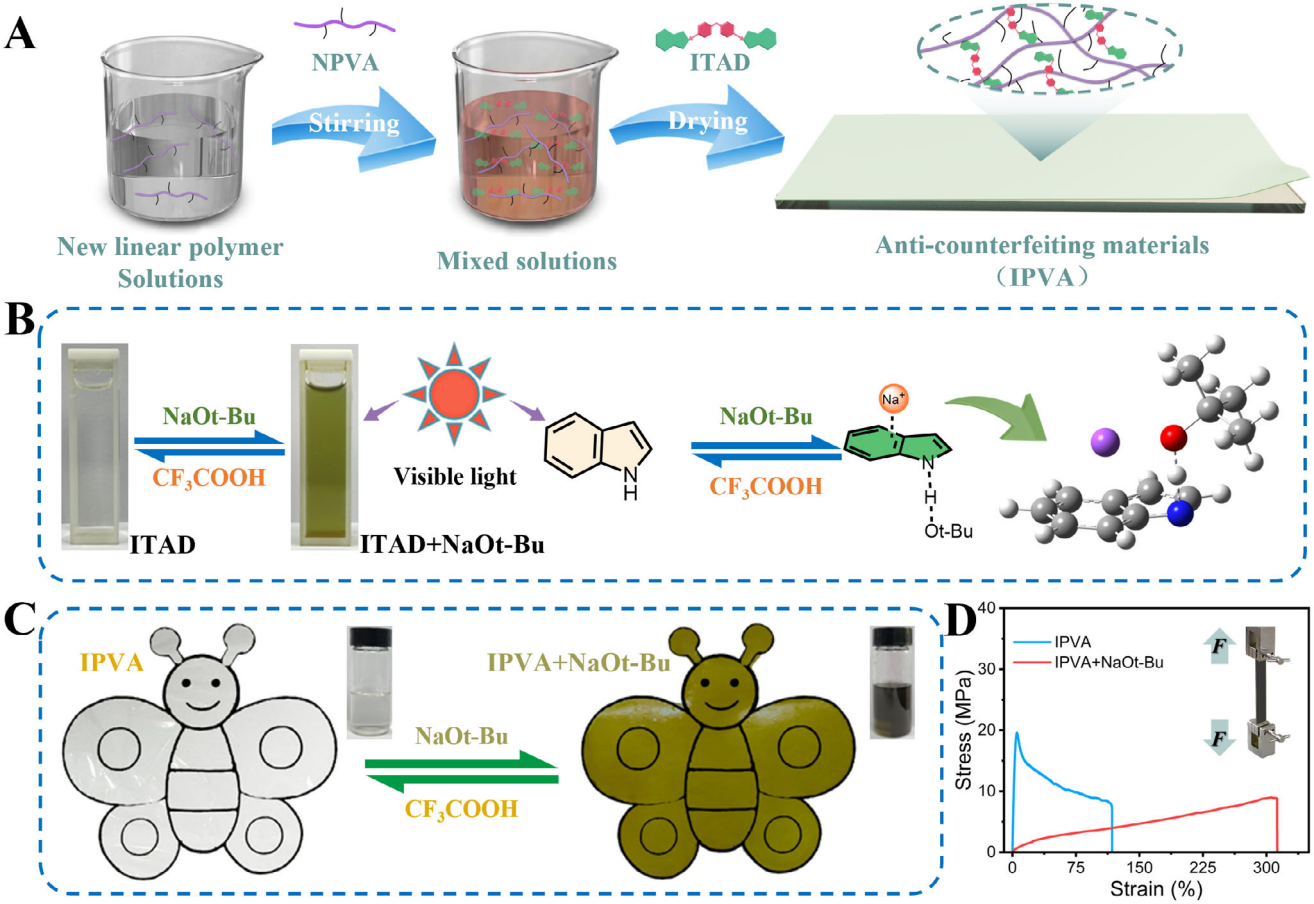
Anti‐counterfeiting characteristics. A) Preparation process of IPVA. B) Structural and color changing of indole groups by introducing NaOt‐Bu and CF_3_COOH. C) Color change images of IPVA films under visible light after stimulation in alkaline conditions. D) The variation stress‐strain curves of IPVA films before and after stimulation in alkaline conditions.

PVA is widely applied in plastics but lacks the inherent ability to undergo color changes in response to external stimuli. Due to the unique push‐pull electronic structure, the incorporation of a fluorescent functional moiety, ITAD, could address this limitation by enabling color changes under external stimuli, thereby making it a promising candidate for anti‐counterfeiting. Herein, we modify the traditional PVA by integrating ITAD into an NPVA system via physical doping (**Figure** **4**A). The resulting IPVA material exhibits high‐contrast color‐switching behavior upon stimulation, ascribed to the protonation‐deprotonation‐induced structural transitions of imine groups under basic and acidic conditions, respectively (Figure 4B). This mechanism is consistent with our prior findings.^[^ ^39^ ^]^ To elucidate the color‐changing mechanism of IPVA under acid‐base stimulation, Gaussian software is employed to calculate the optimized energy and the variations in HOMO and LUMO energy levels of the model compound in its nitrogen anion state (Figure S12, Supporting Information). The results reveal a reduced energy gap between HOMO and LUMO leads to lower‐energy photon absorption and emission, providing theoretical support for the stimuli‐responsive color change. Experimentally, dissolving ITAD in DMF and adding an equivalent amount of NaOt‐Bu caused the solution to change rapidly from pale yellow to bright green (Figure 4B). Adding CF_3_COOH restores the color immediately. Additionally, as shown in Figure 4C, IPVA films doped with just 1% ITAD appear pale yellow under visible light and shift dramatically to green upon the addition of NaOt‐Bu, indicating rapid responsiveness to acid‐base stimuli. Notably, the reaction speed increases with higher ITAD concentrations. The mechanical properties of IPVA films doped with ITAD are also investigated to assess the impact of doping on the polymer matrix. The stress‐strain curves depict a significant enhancement in elongation at break for IPVA+NaOt‐Bu films, accompanied by a slight reduction in tensile strength (Figure 4D). This improvement is attributed to the formation of cation‐π interactions between indole groups, which creates micro‐crosslinking networks within the matrix, consistent with our previous studies.^[^ ^40^ ^]^ This can be observed from the UV and fluorescence spectra before and after alkali addition(Figures S23 and S24, Supporting Information). The UV spectrum clearly shows a pair of intensity variations, characterized by negative intensity at 328 nm and positive intensity at 299 nm, corroborating the stable cation‐π interaction between Na^+^ and the indole ring.^[^ ^41^ ^]^ Upon curing with NaOt‐Bu, the fluorescence intensity exhibited a significant decrease in the IPVA film due to the form of the “point‐face” cation‐*π* interactions (Figure 2D).^[^ ^42^ ^]^ To further understand the stability of the recycled anti‐counterfeiting materials during use, we conducted a long‐term stability test on the anti‐counterfeiting materials, and the results show that the IPVA has good stability (Figure S25, Supporting Information).

## 3 Conclusion

In summary, the **Bio‐Tailoring** technology of thermosets proposed in this work enables a combination of exceptional resource recovery and environmental protection. It is amazing that the green recycling behavior of thermosets driven by microorganisms diverges notably from conventional recycling methods and breaks the recycling bottleneck of thermosets, which cannot be achieved by previously reported physical, chemical, and biological recycling technology. According to the results and discussion described above, the key innovation of this strategy includes two aspects: 1) A new low‐energy consumption recycling method is established for thermosets because the indole groups in the cross‐linked network can be accurately identified and detached by microorganisms; 2) A novel anti‐counterfeiting filler is constructed due to the unique fluorescent functional units that can be produced from thermosets via **Bio‐Tailoring** technology. Taken together, the **Bio‐Tailoring** technology effectively addresses the longstanding paradox surrounding the recyclable and reuse properties of thermosets.

## Conflict of Interest

The authors declare no conflict of interest.

## Supporting information



Supporting Information

## Data Availability

The data that support the findings of this study are available from the corresponding author upon reasonable request.
