# Supplementary material for: Breaking the Recycling Bottleneck of Thermosets via Bio‐Tailoring Technology
Source: Adv Sci (Weinh). 2025 May 8;12(27):2501617. doi: 10.1002/advs.202501617 (PMC12279215; doi:10.1002/advs.202501617)
Supplement: Supplementary file 1 — Supporting Information [file ADVS-12-2501617-s001.docx]

Supporting Information

Breaking the Recycling Bottleneck of Thermosets via Bio-Tailoring Technology

Jinping Yu†,^1^ Huan Wang†,^2^ Yang Xu†,^1^ Boutrous Wemegah,^1^ Yang Lan,^2^ Jing Li,^3^ Li Yang,^1,4*^ Guanjun Chang^1,4*^

**Materials and Methods**

Materials Tryptophol (Tryp), isophorone diisocyanate (IPDI), and polyvinyl alcohol (PVA) were all procured from Shanghai Aladdin Bio-Chem Technology Co., Ltd., and utilized as received. MDI-TAD was synthesized in-house. Soluble starch medium and Kovacs’s indole kit were procured from Qingdao Hi-tech Industrial Park Hope Bio-technology Co., Ltd., and utilized as received. Glucose was procured from Tianjin Zhiyuan Chemical Reagent Co., Ltd., and utilized as received.

Fourier Transform Infrared Spectroscopy (FTIR) FTIR spectra were measured as KBr pellets on a Nicolet 6700 FTIR spectrometer equipped with a deuterated triglycine sulfate (DTGS) detector and a Ge/KBr beamsplitter. Data collection was performed by Thermos Nicolet, version 7.0, in an OMNIC operating system that connects to the spectrometer. FTIR spectra were recorded from 32 scans at a resolution of 4 cm^−1^ in the 4000-500 cm^−1^ range. The final spectra were subtracted by an air spectrum to remove the background.

^13^C/^1^H Nuclear Magnetic Resonance (^13^C NMR/^1^H NMR) ^13^C NMR/^1^H NMR spectra were recorded on a Bruker AMX600 MHz NMR spectrometer within DMSO-*d*_6_, depending on the solubility of the material in these solvents. The chemical shift is represented in δ (ppm) with SiMe_4_ as the internal standard at room temperature.

Differential Scanning Calorimetry (DSC) DSC measurements were carried out on a PerkinElmer DSC-7 calorimeter. Samples were sealed into a standard aluminum pan, and a sealed empty pan was used as a reference. The scan was carried out in a nitrogen atmosphere at a flow rate of 200 mL/min over a temperature range of 30-150^o^C at a scanning rate of 5^o^C/min.

Tensile Testing Uniaxial tensile testing was carried out on an electromechanical universal tester machine (MTS, CMT4304) with a 50 N sensor at a strain rate of 20 mm/min and a temperature of 25^o^C. The dimensions of the casting film samples: are 50.00 mm × 10.00 mm × 0.12 mm. The stress at break and elongation at break were obtained from the stress-strain experiment with at least three identical specimens and reported as averaged values.

Fluorescence Emission Spectrum The fluorescence emission spectrum including in situ relaxation-RF spectrum, was performed by using an RF-6000 spectrophotometer. The emission spectra (300-500 nm) were recorded using an excitation wavelength of 365 nm and 5 nm (excitation)/3 nm (emission) slit widths. The stretching sample and the simple fixture device were directly placed in the sample pool to test the in situ relaxation-RF spectrum.

Ultraviolet/Visible Light (UV/vis) Absorption Spectroscopy UV/vis absorption spectra were measured in absorption mode using UV-2600, which was equipped with an integrating sphere when testing polymer films. Solid UV/vis absorption spectra were measured by placing a polymer-coated quartz sheet in the passageway of incident light using a quartz sheet as the background. Both of stretching sample and simple the fixture device were directly placed in the passageway of incident light to test the relaxation-UV/vis absorption spectra in situ. About 20 um thick films were prepared by a spin coating method. Converting transmission-wavenumber to adsorption-wavenumber is according to the Beer-Lambert law.

Thermogravimetric analysis (TGA) TGA was conducted using a TGA 2/ SF/ 1100 instrument at a heating rate of 10^o^C/min under a nitrogen atmosphere, with a temperature range of 25-800^o^C.

Dynamic Mechanical Analysis (DMA) Thermo-mechanical properties were measured on Q800 (TA Instruments) working in the tensile mode. The sample dimensions were 1 × 0.7 × 0.1 cm^3^. Tests were performed under an isochronal condition at 1 Hz and the temperature was varied between 25^o^C and 200^o^C at a heating rate of 5 ^o^C min^-1^.

**Experimental Procedure**

Synthesis and Characterization of ITr As shown in Figure S1, Under an inert atmosphere, 10 mmol of IPDI was dissolved in 20 mL of dichloromethane. With stirring at 500 rpm, a mixture of Tryp (10 mmol) and dibutyltin dilaurate (DBTDL, 3 wt.%) in dichloromethane was slowly added dropwise to the solution. After the completion of the addition, the temperature was raised to 45°C, and the reaction was allowed to proceed for 4 hours. The progress of the reaction was monitored by thin-layer chromatography (petroleum ether: ethyl acetate = 2:1, R_f_ = 0.5). Upon completion of the reaction, the yellow oily substance was obtained by concentration in a vacuum. Purification was achieved via column chromatography, with a solvent system of petroleum ether: ethyl acetate = 2:1 and an R_f_ value of 0.35. The removal of solvents under reduced pressure yielded a pale-yellow powdered product (2.761 g, 7.2 mmol) with a yield of 72%. The chemical structure of ITr was characterized by FTIR (Figure S2), ^1^H NMR spectra (Figure S3) and ^13^C NMR spectra (Figure S4). The FTIR spectrum of as-synthesized ITr in Figure S2 shows intense absorption bands at 3410 (-OH and -NH stretching), 2277 (-NCO stretching), 1700 (C=O stretching), 1600-1450 (skeleton vibration of indole ring), and 740 cm^-1^ (the characteristic C-H out-of-plane deformation vibration peak in the benzopyrrole structure of indole). The ^1^H NMR spectrum of assynthesized ITr in DMSO-*d*_6_ at 600 MHz is shown in Figure S3: δ = 10.83 (s, 1H), 7.54 (d, J = 7.9 Hz, 1H), 7.34 (d, J = 8.3 Hz, 1H), 7.17 (s, 1H), 7.07 (t, J = 7.2 Hz, 1H), 7.04 (s, 1H), 6.98 (t, J = 8.3 Hz, 1H), 4.17 (t, J = 6.2 Hz, 2H), 3.65 (d, J = 7.9 Hz, 1H), 3.09 (s, 2H), 2.97 (t, J = 7.2 Hz, 2H), 1.52 (q, J = 12.2 Hz, 2H), 1.02 (d, J = 6.2 Hz, 4H), 0.97 (s, 3H), 0.90 (s, 3H), 0.89 (s, 3H) ppm. ^13^C NMR (600 MHz, DMSO-*d*_6_): δ (ppm) = 155.893 (C), 136.245 (C), 127.594 (C), 122.078 (CH), 122.055 (C), 121.815 (CH), 119.431 (CH), 118.833 (CH), 112.238 (C), 111.17 (CH), 64.865 (CH2), 57.012 (CH_2_), 46.621 (CH_2_), 46.081 (CH_2_), 44.587 (CH_2_), 41.568 (CH), 36.537 (CH_3_), 34.944 (CH_3_), 27.539 (CH_2_), 26.854 (CH_3_), 25.264 (C), 23.379 (C).


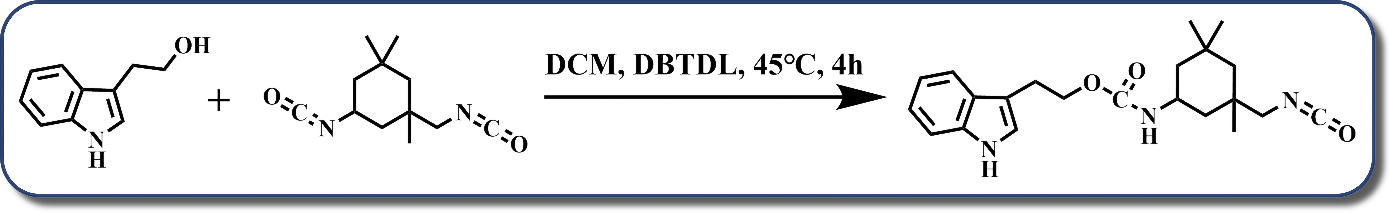


**Figure S1.** Preparation of ITr.


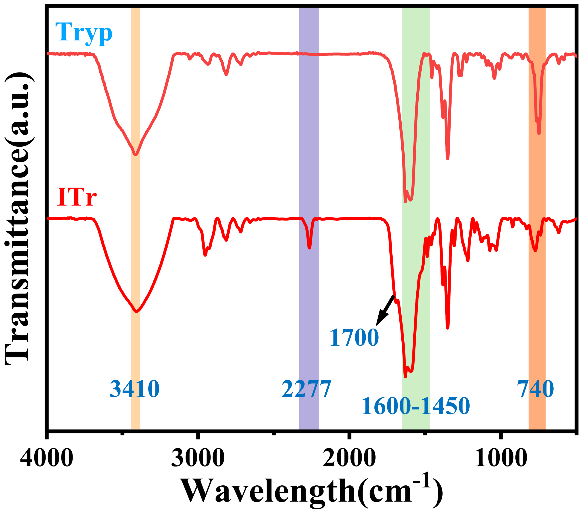


**Figure S2.** The FTIR spectra of ITr.


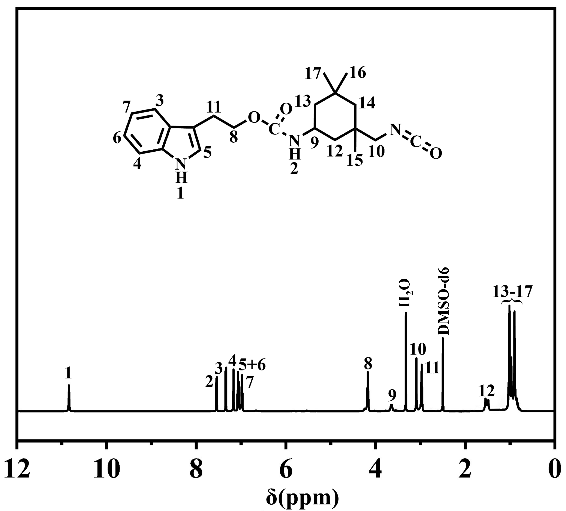


**Figure S3.** The ^1^H NMR spectra of ITr.


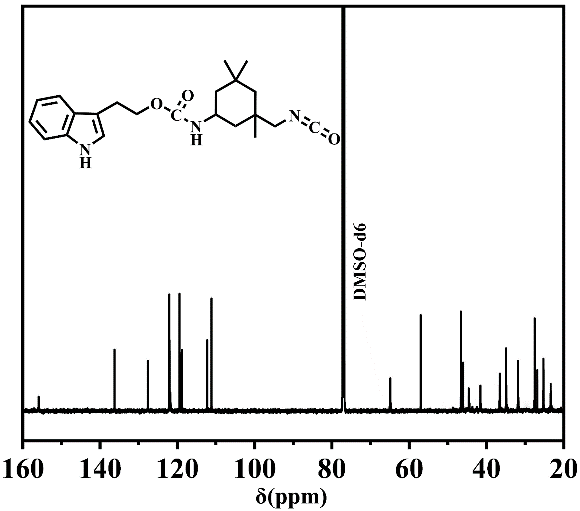


**Figure S4.** The ^13^C NMR spectra of ITr.

Synthesis and Characterization of MDI-TAD As shown in Figure S5, the synthesis of MDI-TAD was carried out following the previously described procedure.^[1]^ A mixture of ethyl carbamate (40.0 g, 0.384 mol, 2 eq) and toluene (300 mL) was placed in a three-neck flask (1 L) and cooled in an ice bath. The flask was equipped with an addition funnel, containing 48.0 g of 4,4’-Methylenebis(phenyl isocyanate) (0.192 mol, 1 eq) dissolved in 200 mL of toluene, a mechanical stirrer, and a bulb condenser. The mixture was put under an inert atmosphere and the isocyanate was added slowly under vigorous stirring. After addition the mixture was stirred at room temperature for 2 hours, followed by 2 hours at 90°C. After cooling the reaction to room temperature, the desired product was filtered off and washed with toluene. In a 1 L flask, bisfunctional semicarbazide (86.2 g, 0.188 mol) was dissolved in 330 mL of an aqueous potassium hydroxide solution (5M) under an inert atmosphere. This mixture was refluxed for 1.5 hours (100°C), warm filtered, cooled to room temperature, and acidified until pH 1 due to the addition of hydrogen chloride. This mixture was cooled to room temperature to yield a solid white powder that was filtered off. A mixture of bisfunctional urazole (2 g, 5.46 mmol, 1 eq), DABCO-Br (5 g, 3.18 mmol, 0.58 eq), and dichloromethane (30 mL) was put in a flask (100 mL) under inert atmosphere and stirred for 2 hours at room temperature. The reaction mixture was filtered off, the residue washed with dichloromethane (2 × 30 mL), and the filtrate was concentrated in vacuo to obtain 4,4’-(4,4’-diphenylmethylene)-bis-(1,2,4-triazoline-3,5-dione). The temperature of the heating bath cannot exceed 50°C due to the volatility of the obtained compound. The chemical structure of MDI-TAD was characterized by ^1^H NMR spectra (Figure S6).


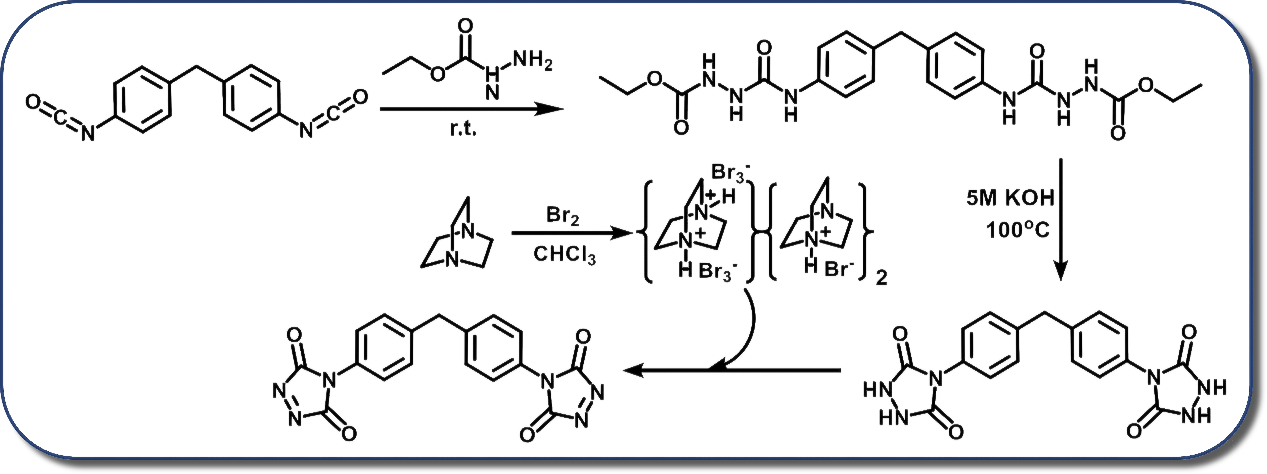


**Figure S5.** The preparation route of 4,4’-(4,4’-diphenylmethylene)-bis-(1,2,4-triazoline-3,5-dione) (MDI-TAD).^[1]^


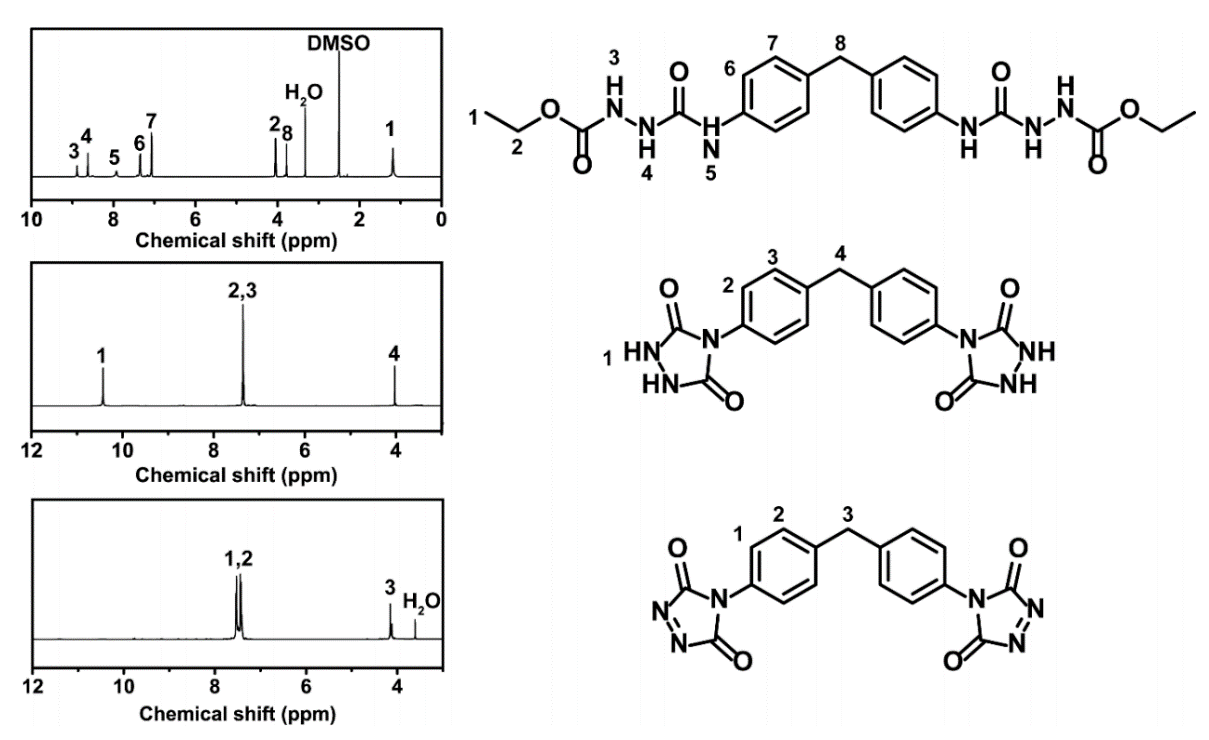


**Figure S6.** ^1^H NMR (in DMSO-*d*_6_) of intermediate 4,4’-(4,4’-diphenylmethylene)-bis-(carbethoxysemicarbazide), 4,4’-(4,4’-diphenylmethylene)-bis-(urazole) (MDI-bisurazole), and the final 4,4’-(4,4’-diphenylmethylene)-bis-(1,2,4-triazoline-3,5-dione) (MDI-TAD) cross-linker.^[1]^

Synthesis and Characterization of Indole-Functionalized Poly(vinyl alcohol) (PIVA) As shown in Figure S7, The synthesis of indole-functionalized poly (vinyl alcohol) (PIVA), with an indole grafting rate of 3%, is described as follows: under an inert atmosphere, 10 mmol of PVA was dissolved in 20 mL of DMSO. The mixture was stirred at 500 rpm and heated to 45°C until the PVA was completely dissolved. Subsequently, 0.4 mmol of ITr and 3 wt.% of DBTDL were added to the solution. The mixture was further stirred at 45°C for 4 hours, and the reaction was monitored by thin-layer chromatography (petroleum ether: ethyl acetate = 2:1, R_f_ = 0.5) until ITr was no longer detectable in the reaction vessel. After the reaction, the mixture was directly poured into a beaker containing dichloromethane, causing the polymer to precipitate and suspend in the solvent. The precipitated polymer was filtered out and dried at 45°C in a vacuum oven. Acetone was used as the solvent for Soxhlet extraction to remove impurities from the polymer over 2 days. After the extraction, the product was dried at 45°C in a vacuum oven to obtain PIVA. The chemical structure of PIVA was characterized by FTIR (Figure S8) and ^1^H NMR spectra (Figure S9). A FTIR spectrum of PIVA is shown in Figure S8 with characteristic absorption bands at 3460, 2990, 2277, 1660, 1600, 1450, 1060, and 740 cm^-1^. ^1^H NMR of PIVA is presented in Figure S9: δ = 10.83 (s, 1H), 7.54 (d, J = 7.9 Hz, 1H), 7.34 (d, J = 8.3 Hz, 1H), 7.17 (s, 1H), 7.07 (t, J = 7.2 Hz, 1H), 7.04 (s, 1H), 6.98 (t, J = 6.0 Hz, 2H), 4.22/4.47/4.67 (s, 1H), 4.16 (t, J = 6.0 Hz, 1H), 3.90 (s, 2H), 3.84 (s, 1H), 3.80 (s, 1H), 2.96 (t, J = 12 Hz, 2H), 2.72 (s, 2H), 1.99 (s, 2H), 1.45 (d, J = 6.0 Hz, 2H), 1.34 (s, 2H), 0.97 (d, J = 12 Hz, 3H), 0.90 (s, 6H) ppm.


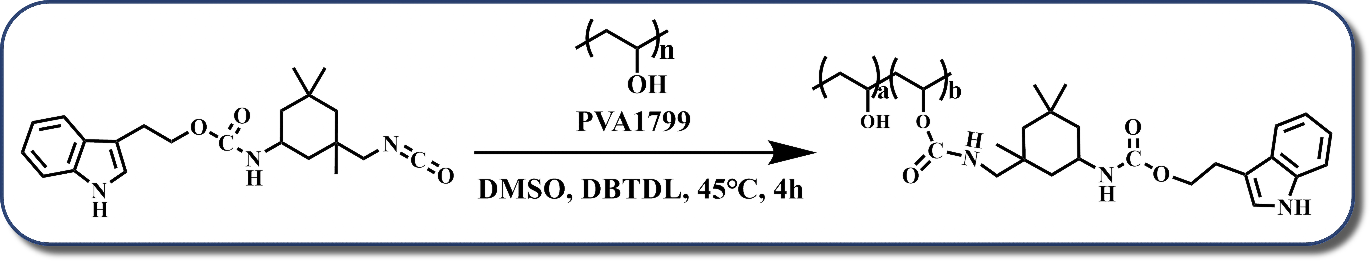


**Figure S7.** Preparation of PIVA.


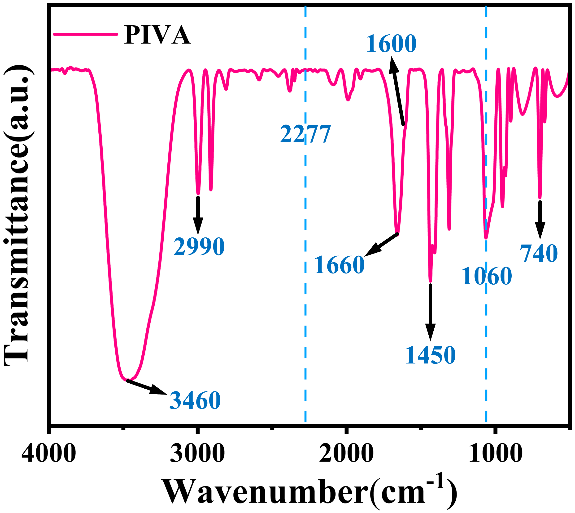


**Figure S8.** The FTIR spectra of PIVA.


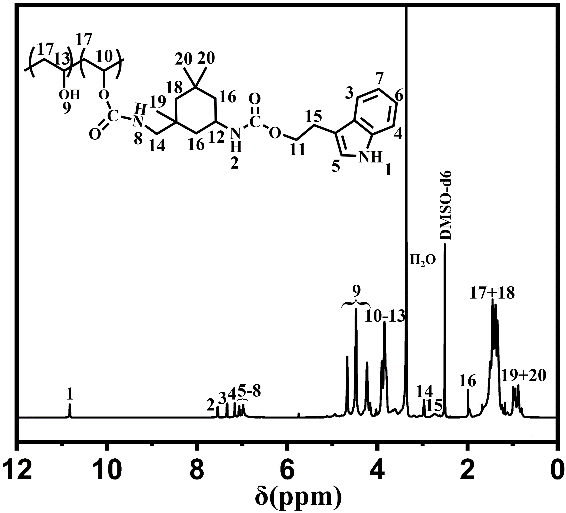


**Figure S9.** The ^1^H NMR spectra of PIVA.

Construction of cross-linked Poly(indole vinyl alcohol) polymer (CPIVA) As shown in Figure 1B, Using a cross-linking density of 12.5% as an example, the construction of cross-linked poly(indole vinyl alcohol) polymer (CPIVA) is described as follows: PIVA (1 g) was completely dissolved in DMSO (15 mL) at 80°C for approximately 2 hours. The polymer solution was then placed in a refrigerator to lower the temperature of the solution to around 15°C, slowing down the cross-linking process during mixing. MDI-TAD (8 mg) was dissolved in a small amount of DMAc solvent and pre-cooled in the refrigerator. The polymer solution was placed on a stirrer at a high stirring speed (1000 rpm). The MDI-TAD solution was poured into the polymer solution, and the resulting red solution was stirred for 20 seconds. Subsequently, the solution was cast onto glass slides, and an organic-solvent transition was observed within half a minute. The solvent was evaporated in a vacuum oven at 80°C for 12 hours to obtain the desired polymer films (approximately 0.05 mm thick) (Figure S10).


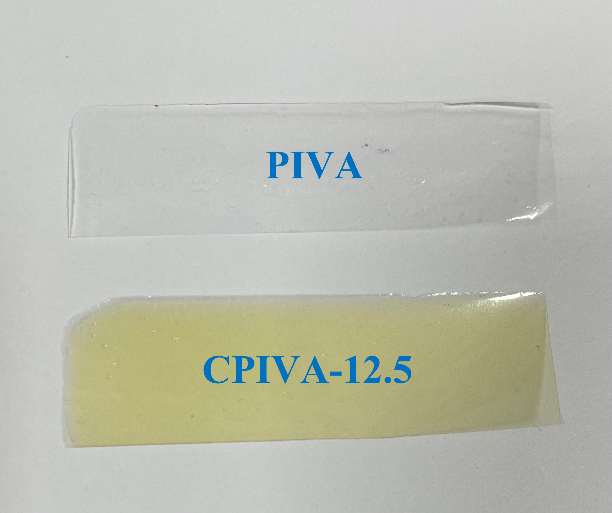


**Figure S10.** The photos of the PIVA and CPIVA-12.5.

Preparation of E. coli Liquid Medium 0.85 g soluble starch medium, 0.5 g glucose, and 50 mL distilled water were weighed in a 100 mL beaker. After stirring and boiling, continue to boil for 10 minutes, cool to room temperature, and then put into a 250 mL conical flask to obtain an unsterilized glucose-starch culture solution. The conical bottle was sealed with cotton gauze and put into a vertical high-pressure steam sterilization pot. The autoclave was set at 121°C for 20 minutes for high-pressure steam sterilization. After the sterilization was completed, it was cooled to room temperature taken out, and transferred to an ultra-clean bench to inoculate E. coli. Put the inoculated liquid medium into a constant temperature incubator for culture and reserve.

General Procedure of Positive Indole Reaction In a typical experiment, 2 mL of E. coli liquid culture solution soaked after tailored CPIVA plastic was placed in a small glass bottle. Then 2-3 mL ether was added to the glass bottle, and indole was extracted into ether by sufficient oscillation. After standing for a while, the ether layer floated on the top of the culture medium. At this time, 3-5 drops of Kovacs’s indole kit were slowly added along the wall of the bottle (do not shake the glass bottle after adding the reagent). If there was indole, the ether layer showed rose red, which was the positive indole reaction. In contrast, the ether layer showed yellow or none, which was negative. This is the result of Microbiological Quality Control provided by Qingdao Hi-tech Industrial Park Hope Bio-technology Co., Ltd (**Figure S11**).


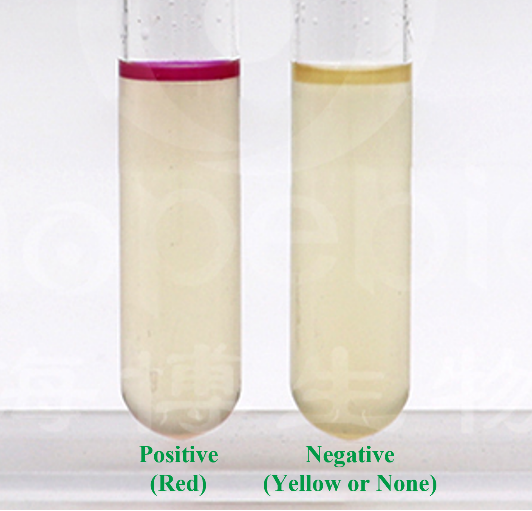


**Figure S11.** Photo of *Microbiological Quality Control* results.

**Density Functional Theory (DFT) Calculation**

Method All computation was carried out with the Gaussian 16 Revision A.03 software package. The Berny algorithm was used to locate stationary points. Different conformations are set by increasing the distance between the ion and indole plane. Very tight convergence criteria and ultrafine integration grids were used in all optimizations. The calculations of analytical frequencies on converged constrained molecules are valid because the molecule plus its infinitely-compliant constraining potential is a stationary point.^[2]^ To start with, the B3LYP/6-31+G(d, p) level of theory was used for geometry optimizations.^[3]^ The B3LYP functional has been proven to produce good geometries but is less accurate for energy calculations. Therefore, energies were refined with the M06-2X functional,^[4, 5]^ which can account for dispersion effects, and a 6-31++G(d, p) basis set.


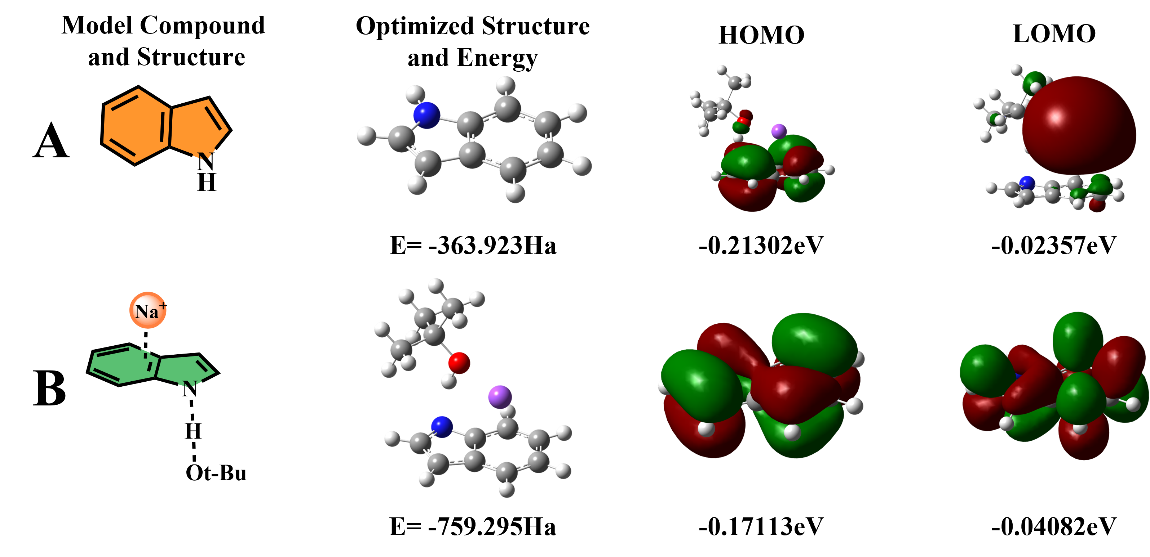


**Figure S12.** Model compound structure and corresponding Gaussian calculation results.

**Solubility of** **PIVA and CPIVA-12.5**

The solvent resistance is one of the key properties of thermosets. In this part, we examined the solubility of PIVA and CPIVA-12.5. As shown in Table S1, the CPIVA-12.5 plastic has a stable structure and excellent solvent resistance.

**Table** **S1.** Solubility of the thermoset plastic in different solvents.

| Sample | DMAc^a^ | DMSO^b^ | DMF^c^ | NMP^d^ | THF^e^ |
| --- | --- | --- | --- | --- | --- |
| PIVA | +^e^ | + | + | + | - |
| CPIVA-12.5 | - | - | - | - | - |

^a^*N,N*-Dimethylacetamide (DMAc); ^b^Dimethyl Sulfoxide (DMSO); ^c^*N,N*-Dimethylformamide (DMF); ^d^*N*-Methyl-2-pyrrolidone (NMP); ^e^Tetrahydrofuran (THF); +: the polymer can be completely dissolved at room temperature; -: the polymer was insoluble at room temperature.

**Effect of the Proportion of MDI-TAD on the Mechanical Properties**

In this part, we investigated the effect of the feed ratio for MDI-TAD on the mechanical properties of CPIVA. The fraction of MDI-TAD is varied from 0 % to 100 %. Representative stress-strain curves are shown in Figure S13. Overall, the mechanical properties of the CPIVA plastics with varying MDI-TAD proportions are much greater than those of PIVA films. As illustrated in Figure S13, the extensibility and tensile strength of the thermoset plastics exhibit an increase with the addition of MDI-TAD up to 12.5%, followed by a decline when the concentration rises from 12.5% to 15%. When the crosslinking density further increases, the performance of the material decreases rapidly (Figure S14). The CPIVA becomes a completely brittle material when it reaches 100% crosslinking. This is because when the crosslinking density increases, the molecular chains inside the material are tightly connected into a dense network, which makes the movement ability of the molecular chain segments significantly limited. Because the chain segment cannot move freely, the material cannot dissipate energy through the slip or rearrangement of the chain segment when subjected to external forces, resulting in energy concentration in the local area and forming a stress concentration point. Meantime, the high crosslinking density makes the glass transition temperature of the material increase, and the material is more likely to be in a glassy state at room temperature, showing higher brittleness. Uniaxial tensile tests reveal an obvious enhancement in the mechanical properties of the CPIVA plastics due to the covalent crosslinking. This room-temperature fast-click crosslinking method provides a controllable crosslinking and simple operation method for constructing thermoset plastics while ensuring the mechanical properties of the materials. Moreover, the retention of the UV absorption peak before and after crosslinking confirmed that TAD first undergoes a click reaction with the C2-position of indole and does not destroy the aromaticity of indole (Figure S15).


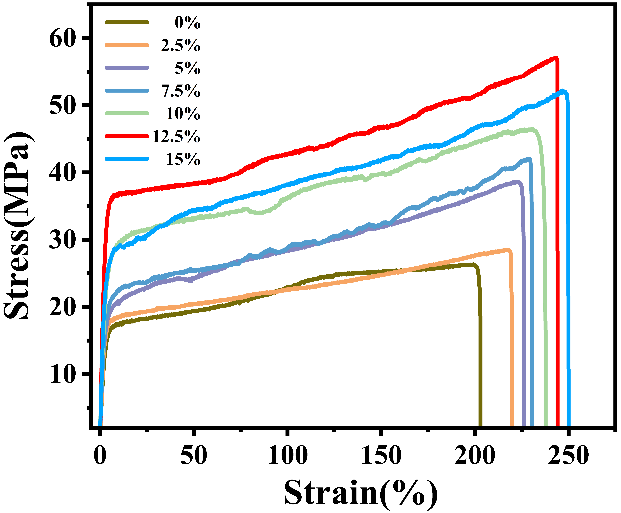


**Figure S13.** Mechanical performance testing of CPIVA with different MDI-TAD feed ratios.


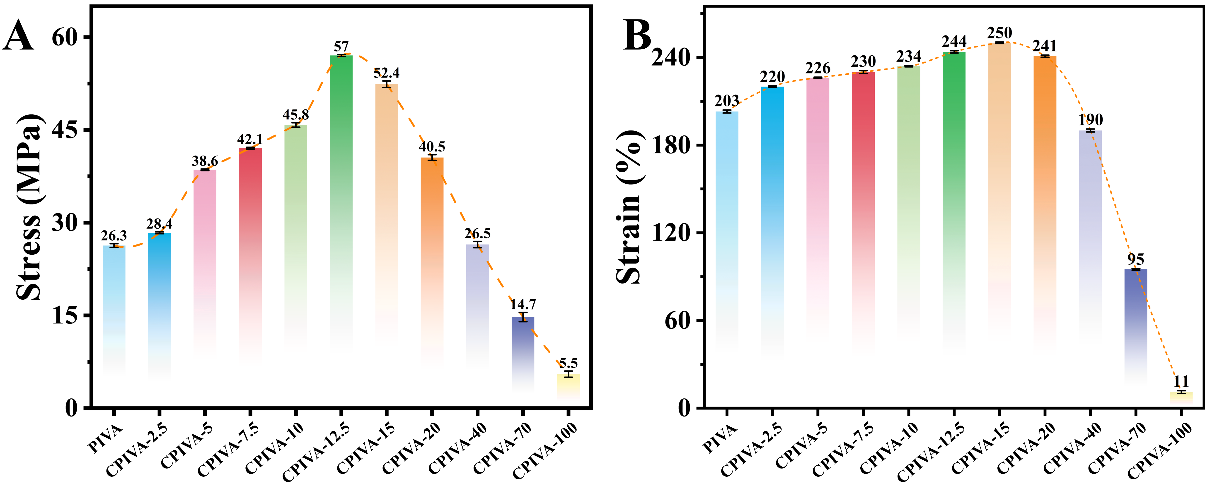


**Figure S14.** Mechanical performance testing of CPIVA with different crosslinking density.

**
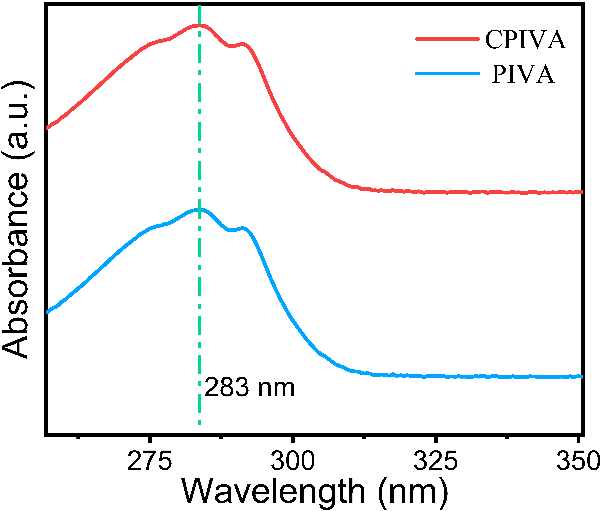
**

**Figure S15.** The UV-visible spectra of PIVA and CPIVA-12.5.

**Thermo Properties of PIVA and CPIVA**

The use of advanced polymers in many practical applications depends on their environmental and thermal stability. In this work, the thermal properties of PIVA and CPIVA plastics were evaluated via TGA (Figure 2D). TGA curves suggest that the plastics are thermally stable up to 260^o^C. DSC of CPIVA-12.5 plastic up to ~90^o^C shows a single T_g_ (Figure S16). In addition, to further understand the overall performance of CPIVA plastics, we carried out experiments on the relationship between crosslinking density, T_g_ and mechanical properties (Figure S17). The results show that with the increase of crosslinking density, the T_g_ and mechanical properties (tensile strength and elongation at break) of CPIVA plastics show a certain change rule. The higher the crosslinking density, the higher the T_g_, and the higher the thermal stability of CPIVA plastics. When the crosslinking density reaches 12.5%, the mechanical properties of CPIVA plastic are optimal. When the crosslinking density further increases, the tensile strength and fracturing toughness of CPIVA plastics decrease significantly. This is because the increase in crosslinking density limits the movement of the polymer chains, resulting in an increase in T_g_, while enhancing the physical crosslinking between the molecular chains, leading to CPIVA plastics showing greater brittleness. Meanwhile, we performed dynamic mechanical analysis (DMA) tests on PIVA and CPIVA-12.5 to evaluate their thermal stability and mechanical properties (Figure S18). The results showed that due to chemical crosslinking, the rigidity and thermal stability of CPIVA-12.5 increased, and CPIVA-12.5 showed twice the storage modulus of PIVA. In addition, because the C-N bond formed between triazolindione (TAD) and indole is a thermally reversible dynamic covalent bond, the C-N bond dissociation of CPIVA-12.5 occurs at 100^o^C.


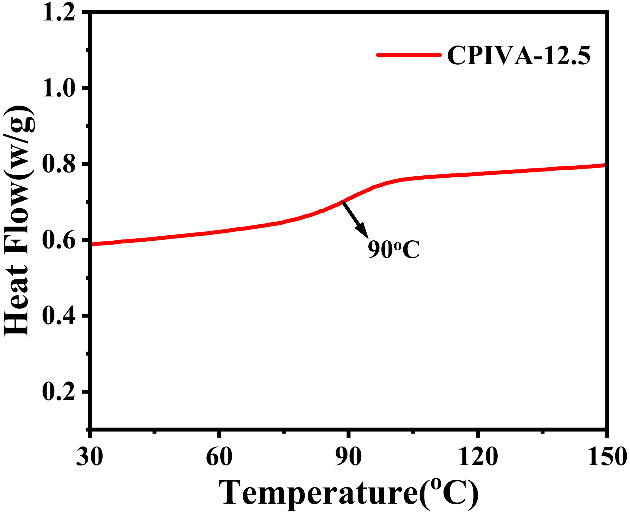


**Figure S16**. The DSC spectra of CPIVA-12.5.


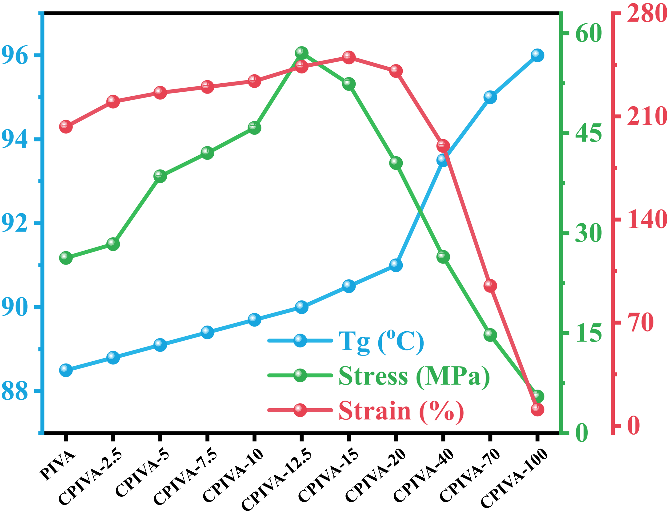


**Figure S17**. The relationship between crosslinking density, Tg and mechanical properties.


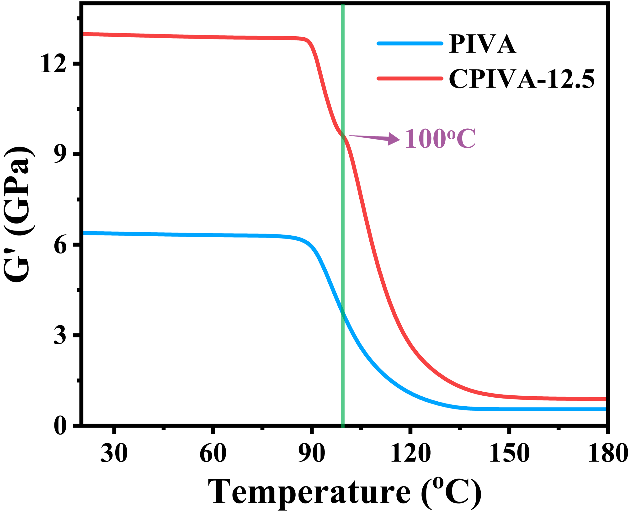


**Figure S18**. The DMA spectra of PIVA and CPIVA-12.5.

**The Nuclear Magnetic Comparison Experiment of Model Compounds**

To further understand the mechanism of ***Bio-Tailoring Technology***, we carried out a nuclear magnetic comparison experiment of ***Tryp***, ***Tryp Compound*** (the extracting solution of liquid medium before ***Bio-Tailoring***) and ***Tailored Compound*** (the extracting solution of liquid medium after ***Bio-Tailoring***). As shown in Figure S19, the ^1^H-NMR spectrum of the extract from the tailored liquid culture medium reveals a new peak at 6.41 ppm, corresponding to the hydrogen on the C3 of the indole ring. The peaks at 4.61 ppm (-OH), 3.65 ppm (-CH_2_-), and 2.84 ppm (-CH_2_-) have disappeared. In contrast, the peaks associated with the hydrogens on the indole ring and the -NH group remain unchanged, appearing near 7 ppm and 11 ppm respectively. These observations indicate the disappearance of Tryp from the liquid culture medium and the concomitant appearance of indole. This transformation demonstrates the precision of Bio-Tailoring Technology in recognizing and tailoring the indole structure.

**
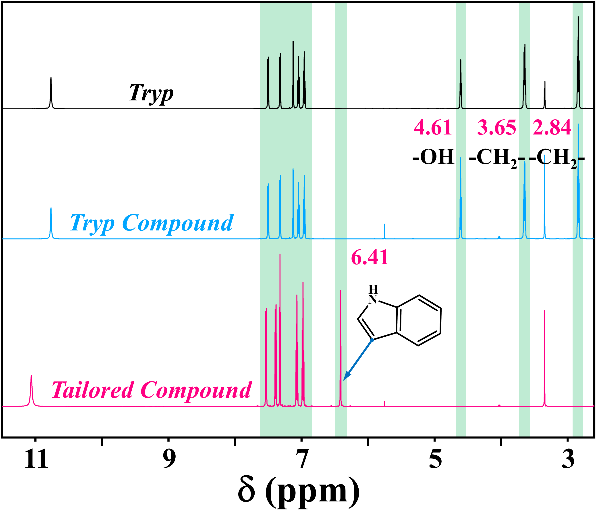
**

**Figure S19**. The ^1^H-NMR spectra of ***Tryp***, ***Tryp Compound*** and ***Tailored Compound***.

**Macroscopic Phenomenon and Positive Indole Reaction of the Liquid Medium**

The positive indole reaction was carried out of the original E. coli liquid medium and had no color change (the result was negative) and showed that there was no indole group in the liquid medium. However, the results of the positive indole reaction in a liquid medium were gradually obvious (rose red gradually increased) with the tailoring process of E. coli (Figure S20). This is because CPIVA-12.5 plastic has many indole groups, and the indole groups gradually fall off with the tailoring process of E. coli, resulting in a significant increase in rose red of the positive indole reaction.

**
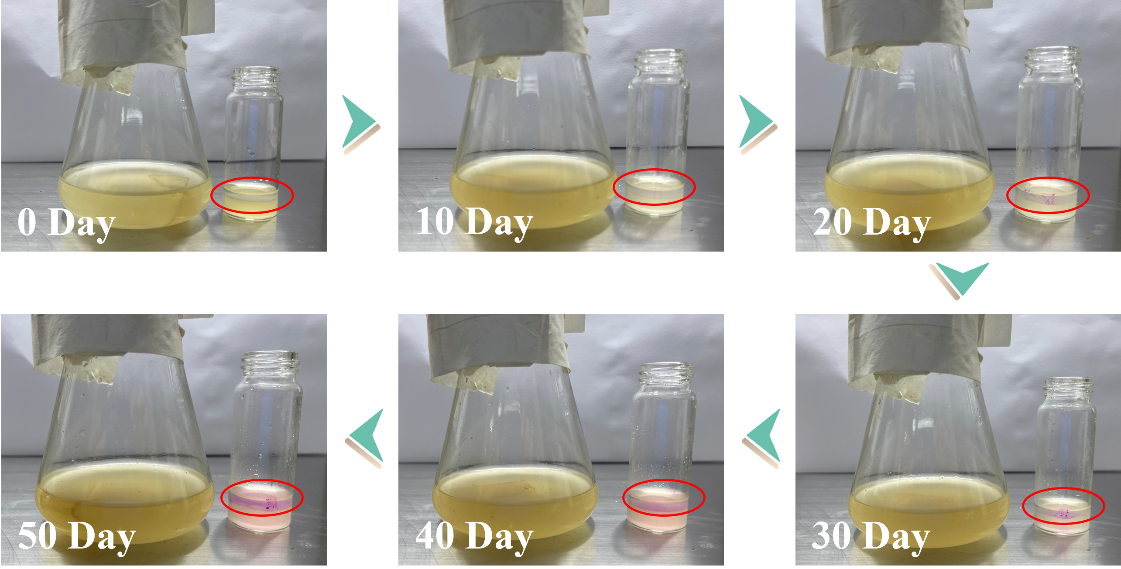
**

**Figure S20.** The phenomenon of indole positive reaction varies with the tailoring time.

**Bio-Recycling of Thermoset Plastics**

The CPIVA-12.5 plastic was insoluble at room temperature and high temperature due to the covalent crosslinking (Figure S21-Ⅰ). However, CPIVA-12.5 plastic can be dissolved in DMSO after being Bio-Tailored by E. coli, indicating the covalent crosslinking network was successfully interrupted (Figure S21-Ⅱ, Ⅲ, Ⅳ, Ⅴ). Surprisingly, the residual plastic dissolved in DMSO could be reconstructed into a film, and the film still maintains an appreciable mechanical property (Figure S21-Ⅵ and S22). The results showed that E. coli only tailors the crosslinking segment of the covalent crosslinking network and does not destroy the main chain structure of the polymer.

**
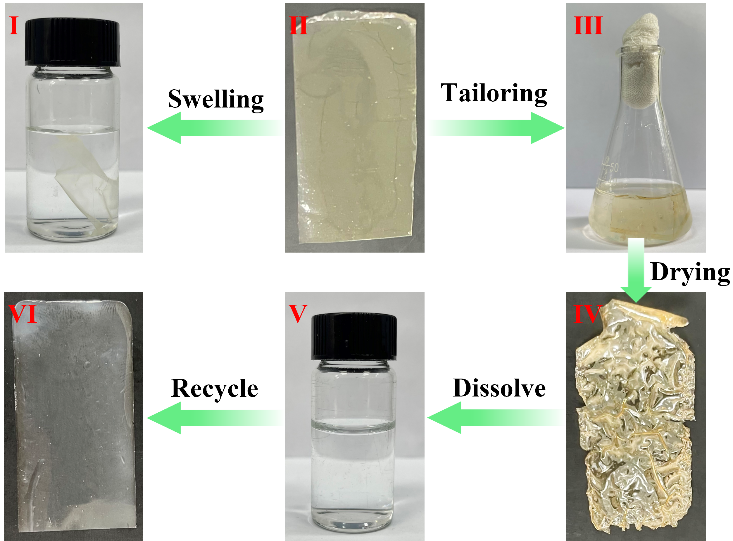
**

**Figure S21.** The ***Bio-Recycling*** process of thermoset plastics.

**
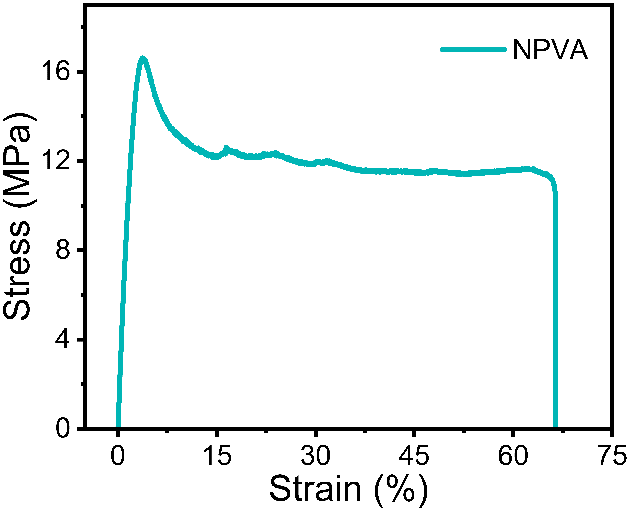
**

**Figure S22.** The mechanical properties of the reconstructed film.

**Proof of Cation-π Interactions Between NaOt-Bu and ITAD**

The UV spectrum clearly shows a pair of intensity variations, characterized by negative intensity at 328 nm and positive intensity at 299 nm, corroborating the stable cation-π interaction between Na^+^ and the indole ring.^[6]^ Upon curing with NaOt-Bu, the fluorescence intensity exhibited a significant decrease in the IPVA film due to the form of the “point-face” cation-π interactions (Figure 2D).^[7]^

**
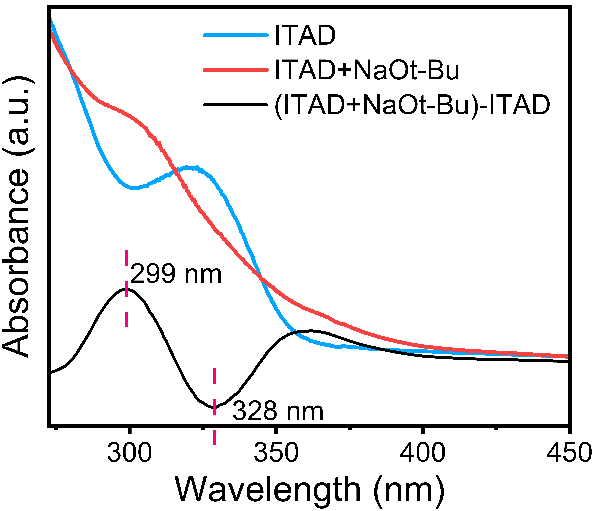
**

**Figure S23.** The UV-visible spectra of ITAD and ITAD+NaOt-Bu.

**
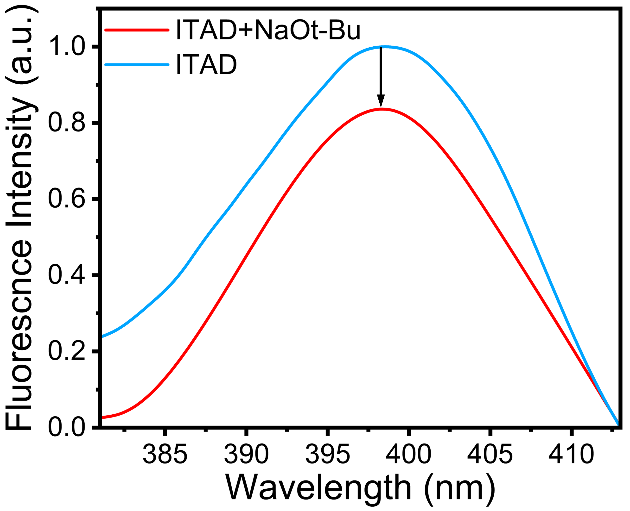
**

**Figure S24.** The Fluorescence spectra of ITAD and ITAD+NaOt-Bu.

**The long-Term Stability of Anti-Counterfeiting Materials**

To further understand the stability of the recycled anti-counterfeiting materials during use, we conducted a long-term stability test on the anti-counterfeiting materials (Figure S25). One year ago, we successfully prepared new plastic packaging materials and anti-counterfeiting small molecule fragments and then placed them in the environment (Mianyang is located in the northwest of the Sichuan Basin, which belongs to the subtropical humid monsoon climate zone. The annual average temperature is about 16~18°C, and the annual precipitation is about 800~1000 mm. The relative humidity of the air varies greatly. The summer humidity is high, up to 70~80%, and the winter humidity is low, about 40~50%. The test site is representative and can reflect the stability performance of the material in the actual application environment). After one year of stability test, the new plastic packaging materials (Figure S25B) and anti-counterfeiting small molecule fragments (Figure S25A) showed obvious acid-base stimulation discoloration phenomenon and had stable anti-counterfeiting performance. In addition, the mechanical properties of IPVA were characterized. The results show that the mechanical properties of IPVA do not change significantly during long-term service, and it has good stability (Figure S25C).


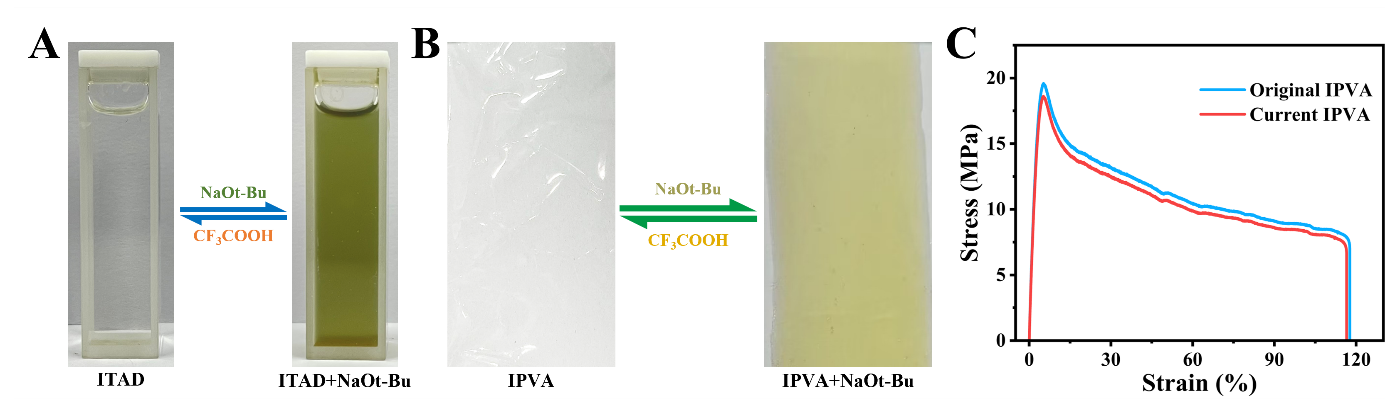


**Figure S25.** The long-term stability of anti-counterfeiting materials.

**References**

1. M. Du, H. A. Houck, Q. Yin, Y. Xu, Y. Huang, Y. Lan, L. Yang, F. E. Du Prez, G. J. Chang, *Nat. Commun.* **2022**, 13, 3231.
2. T. J. Kucharski, R. Boulatov, *J. Mater. Chem.* **2011**, 21, 8237.
3. C. Lee, W. Yang, R. G. Parr, *Phys. Rev. B* **1988**, 37, 785.
4. Y. Zhao, D. G. Truhlar, *Theor. Chem. Acc.* **2008**, 120, 215.
5. L. P. Liu, D. Malhotra, R. S. Paton, K. N. Houk, G. B. Hammond, *Angew. Chem. Int. Ed.* **2010**, 49, 9132.
6. H. Yorita, K. Otomo, H. Hiramatsu, A. Toyama, T. Miura, H. Takeuchi, *J. Am. Chem. Soc.* **2008**, 130, 15266.
7. L. J. Juszczak, A. S. Eisenberg, *J. Am. Chem. Soc.* **2017**, 139, 8302.
